# Evaluation of the Potential of Brazilian Propolis against UV-Induced Oxidative Stress

**DOI:** 10.1155/2011/863917

**Published:** 2010-09-08

**Authors:** Yris Maria Fonseca, Franciane Marquele-Oliveira, Fabiana T. M. C. Vicentini, Niege Araçari J. C. Furtado, João Paulo B. Sousa, Yara M. Lucisano-Valim, Maria José Vieira Fonseca

**Affiliations:** ^1^Departamento de Ciências Farmacêuticas, Faculdade de Ciências Farmacêuticas de Ribeirão Preto-USP, Ribeirão Preto 14040-903, Brazil; ^2^Departamento de Física e Química, Faculdade de Ciências Farmacêuticas de Ribeirão Preto-USP, Ribeirão Preto 14040-903, Brazil

## Abstract

This study investigated the potential use of topically and orally administered propolis extracts to prevent UV irradiation-induced oxidative stress in skin. The results illustrated that green propolis extract (GPE) contained greater amounts of polyphenols, coumaric acid, drupanin, baccharin and artepillin C than did brown propolis extract (BPE). GPE showed higher antioxidant activity than BPE when the IC_50_ (concentration that caused 50% inhibition) values were compared. Interesting, the oral treatment of hairless mice demonstrated a recovery of 30.0% for GPE and 22.8% for BPE with respect to UV irradiation-induced GSH depletion. The topical pretreatment of animals with both propolis extract solutions recovered around 14.0% of the depleted GSH. However, the employed treatments did not inhibit the increase of cutaneous proteinase secretion/activity caused by irradiation. These findings indicate that despite differences in composition and antioxidant properties, GPE and BPE both successfully prevent UV-induced GSH depletion *in vivo* and are both promising antioxidant systems against oxidative stress in skin. Based on these findings, complementary studies should be performed to enhance our understanding of the protective effects of propolis extracts in skin.

## 1. Introduction

Skin is the largest human organ and the only organ directly exposed to ultraviolet (UV) irradiation. It is well known that reactive oxygen species (ROS) are associated with premature skin aging (photoaging), local and systemic immunosuppression, many cutaneous inflammatory disorders, and photocarcinogenesis [1]. Due to the deleterious effects of ROS in the skin, many researchers have endeavored to identify and evaluate antioxidants to enrich the endogenous cutaneous protection system, to prevent and/or treat UV irradiation-induced skin damage.

Much attention has been paid to antioxidants from natural sources, especially flavonoids [2] and other phenolic compounds [3]. It has been reported that orally and topically administered natural antioxidants, such as flavonoids, carotenoids, ferrulic acid, and superoxide dismutase provide protection against UV irradiation-induced erythema and cytotoxicity [2, 4]. 

Due to their high concentrations of antioxidant compounds, natural extracts, such as propolis extracts, have been tested for their ability to prevent UV-induced skin damage. Propolis has been shown to have antitumoral [5], anti-inflammatory [6], antioxidant [7], radioprotective [8], hepatoprotective [9], imunomodulatory [10], and mainly antibacterial [11] properties in both *in vitro* and *in vivo* studies. 

Therefore, the present study was designed to evaluate the potential applicability of two Brazilian propolis extracts (green and brown) for the prevention of oxidative stress in skin due to UV irradiation. With this aim, the first set of experiments compared the physico-chemical composition and the antioxidant potential of propolis extracts. In the second set of experiments, the *in vivo* capacity of these extracts to prevent UVB irradiation-induced reduced glutathione (GSH) depletion and the secretion/activity of metalloproteinases of hairless mice skin were assessed. Both oral and topical treatments were investigated in these *in vivo* studies.

## 2. Materials and Methods

### 2.1. Chemicals

Brazilian brown propolis extract (BPE) purchased from APIS FLORA (Ribeirao Preto—SP, Brazil) was standardized using propolis from several sites in Brazil (Patent number PI 0405483-0, published in Revista de Propriedade Industrial no. 1778 from 01/02/2005). Brazilian green propolis (GPE) was a gift from Bioessens Ltda (Cotia—SP, Brazil). Both BPE and GPE with 11% dry weight. Luminol, horseradish peroxidase (HRP), thiobarbituric acid (TBA), ethylene glycol bis (-aminoethyl ether)-*N*,*N*,*N*′,*N*′-tetraacetic acid (EGTA), xanthine, and xanthine-oxidase (XOD) were purchased from Sigma Chemical Co. (St. Louis, MO, USA). Hydrogen peroxide (36%) was purchased from Calbiochem (CA, USA), and deoxyribose, *o*-phthalaldehyde (OPT), and quercetin were purchased from Acros (New Jersey, USA). Gallic Acid and Folin-Ciocalteu were purchased from Merck (Darmstadt, Germany). All other chemicals were of reagent grade and were used without further purification.

### 2.2. Physico-Chemical Composition

#### 2.2.1. Total Polyphenol and Flavonoid Content in Brown and Green Propolis Extracts

Total polyphenol and flavonoid content in brown and green propolis extracts were determined by the Folin-Ciocalteau colorimetric and aluminum chloride colorimetric methods, respectively [12].

#### 2.2.2. HPLC Analysis

Propolis samples were subjected to HPLC analysis using a Shimadzu High Performance Liquid-Chromatograph (SCL-10A VP system controller, three LC-10AD pumps, SPD- M10AVP photodiode array detector, and Shimadzu Class-VP (5.02) software. The separation was carried out on a CLC-ODS column (4.6 mm ID × 250 mm, 5 *μ*m particle diameter) and a CLC G-ODS [8] column was used as a guard column. A gradient starting with 0.8% acetic acid, 0.3% ammonium acetate, 5.0% methanol/water, and 25% acetonitrile and finishing with 100% acetonitrile, over 60 minutes (flow rate 1.0 mL/minute), was used to separate the major compounds, which were identified by comparison with authentic standards previously isolated from Brazilian green propolis [13, 14].

### 2.3. Antioxidant Potential

The antioxidant activity of propolis extracts was evaluated by inhibition of lipid peroxidation as described by Marquele and collaborators [15], the H_2_O_2_/luminol/HRP assay [16], scavenging of superoxide radicals produced in the chemiluminescence assay using the xanthine/luminol/XOD system [17], and the deoxyribose assay as described by Halliwell et al. [18].

Five hundred microliters of propolis extract were solubilized in propylene glycol (1 : 10) and diluted using the medium from each reaction to the following final concentration ranges: 0.004–0.1 *μ*L/mL for the lipid peroxidation assay, 0.00006–0.2 *μ*L/mL for the deoxyribose assay, 0.036–0.4 *μ*L/mL for the chemiluminescent assay using the H_2_O_2_/luminol/HRP system, and 0.0013–0.04 *μ*L/mL for the chemiluminescent assay using the xanthine/luminol/XOD system.

For all techniques, the percentage of inhibition was plotted against the concentration of propolis extract and the concentration that caused 50% inhibition of the system was reported as the IC_50_ value [15].

### 2.4. In Vivo Protective Effect against UVB-Induced Oxidative Stress

#### 2.4.1. Animals and Experimental Protocol

*In vivo* experiments were performed on 3-month-old, sex-matched hairless mice of the HRS/J strain. The animals, weighing 20–30 g, were housed in a temperature-controlled room with access to water and food *ad libitum* until use. All experiments were conducted in accordance with National Institutes of Health guidelines for the welfare of experimental animals and with the approval of the Ethics Committee of the Faculty of Pharmaceutical Science of Ribeirao Preto (University of Sao Paulo).

The animals were divided into 8 groups (*n* = 3): 4 groups for topical treatment and 4 groups for oral treatment. The topical treatment experiment featured the following groups: Group 1 = nonirradiated control (propylene glycol treatment), Group 2 = irradiated control (propylene glycol treatment), Group 3 = irradiated and treated with a solution containing 2.5% GPE in propylene glycol, and Group 4 = irradiated and treated with a solution containing 2.5% BPE in propylene glycol. Three hundred microliters of the test solutions were applied on the dorsal side of the animals, 1 hour before and 5 minutes before irradiation. The oral treatment was tested on the following experimental groups: Group 1 = nonirradiated control (water treatment), Group 2 = irradiated control (water treatment), Group 3 = irradiated and treated with a solution of 100 mg/kg of GPE in 30% ethyl alcohol, and Group 4 = irradiated and treated with a solution of 100 mg/kg of BPE in 30% ethyl alcohol. The treatment protocol involved the oral application of 100 *μ*L of the test solutions (30 mg/mL) 18 hours before and 30 minutes before irradiation [19].

#### 2.4.2. Irradiation

The UV source of irradiation consisted of a Philips TL/12RS 40 W lamp (Medical-Holand). This source emits in the range of 270–400 nm with an output peak at 313 nm resulting in an irradiation of 0.27 mW/cm^2^ at a distance of 20 cm, as measured by an IL 1700 radiometer (Newburyport, MA, USA) equipped with an UVB and UV detector. The minimal dose that induces GSH depletion and gelatinase activity (2.46 J/cm^2^) was determined by Casagrande et al. [20]. The mice were killed with an overdose of carbon dioxide 6 hours after the UV exposition, and full dorsal skins were removed and stored at −80°C until analysis.

#### 2.4.3. GSH Assay

The GSH skin levels were determined using a fluorescence assay as previously described by Hissin and Hilf [21]. The total skin of hairless mice (1 : 3 dilution) was homogenized in NaH_2_PO_4_ (100 mM; pH 8.0) containing EGTA (5 mM) using a Turrax TE-102 (Turratec, Sao Paulo). Whole homogenates were treated with 30 % trichloroacetic acid and centrifuged at 1900 *g* for 6 minutes, and the fluorescence of the resulting supernatant was measured with a Hitachi F-4500 fluorescence spectrophotometer.

#### 2.4.4. Qualitative Analyses of Skin Proteinases by Substrate-Embedded Enzymography

SDS-PAGE (sodium dodecyl sulfate polyacrylamide gel electrophoresis) substrate-embedded enzymography (zymography) was used to detect enzymes with gelatinase activity. Assays were carried out as previously reported by Onoue et al. [22] and Vicentini et al. [23]. The proteolytic activity was qualitatively analyzed by comparing control animals and animals treated with propolis extracts. The Lowry method was used to measure protein levels in the skin homogenates [24].

### 2.5. Statistical Analysis

Data are expressed as means ± SE determined from triplicate analyses. The concentration of propolis extracts that caused 50% of inhibition of the system assessed (IC_50_) were determined using GraphPad Prism software. Data were statistically analyzed by the Student's *t*-test, and the level of significance was set at *P* < 0.05.

## 3. Results

### 3.1. Physicochemical Composition

**Figure 1:**
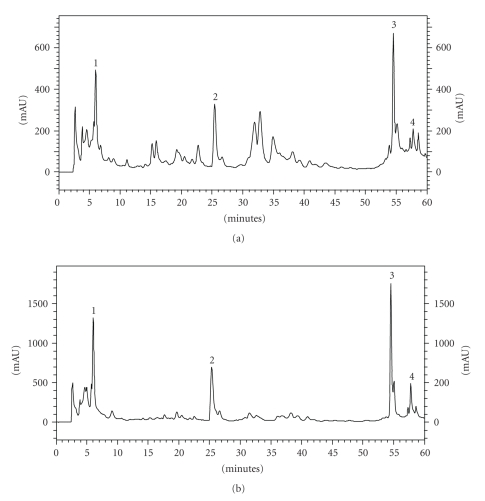
Chromatograms of BPE (a) and GPE (b). 1: *p*-coumaric acid, 2: drupanin, 3: artepillin C, and 4: baccharin.

The total flavonoid and polyphenol contents of both extracts were assessed. The results showed that GPE contained 1.78% and 0.23% of polyphenols and flavonoids, respectively. BPE contained 1.33% and 0.47% of polyphenols and flavonoids, respectively. The HPLC analysis of propolis extracts identified the following major compounds: *p*-coumaric acid, drupanin, artepillin C, and baccharin (Figures 1(a) and 1(b)). While the extracts had similar chemical compositions, the concentration of various antioxidant compounds varied (Table 1). Overall, GPE presented with higher concentrations of the evaluated compounds than did BPE. GPE contained 275.7 *μ*g/mL, 529.5 *μ*g/mL, 1053.7 *μ*g/mL, and 1060.8 *μ*g/mL of *p*-coumaric acid, drupanin, artepillin C, and baccharin, respectively. BPE, however, contained 74.6 *μ*g/mL, 213.6 *μ*g/mL, 477.9 *μ*g/mL, and 585.1 *μ*g/mL of *p*-coumaric acid, drupanin, artepillin C, and baccharin, respectively. Nevertheless, when the extracts were compared at the same dilution and under the same chromatographic conditions, BPE showed more peaks within the retention time range of 10–45 minutes, suggesting a greater diversity of compounds.

### 3.2. Antioxidant Potential

Skin exposure to ultraviolet radiation induces the formation of lipid peroxidation products, lipid radicals, melanin radicals, endogenous antioxidant depletion, and ROS overproduction [20, 25]. Our group has previously demonstrated the potential *in vitro* antioxidant activity of BPE [15]. Additionally, it was suggested that a topical formulation containing BPE might be effective in protecting skin against UVB photodamage, by accelerating the cellular renewal (cicatrization) and inhibiting the inflammatory process [26]. The present study was designed to further characterize the ability of BPE to prevent oxidative stress and to discover if GPE had similar properties. Thus, the antioxidant activity of GPE against different free radicals was evaluated using several antioxidant methods, and the results were compared to the antioxidant activity of BPE. Some of these methods were able to generate several ROS in skin after exposure to UV irradiation, such as superoxide (O^2−^), hydroxyl radicals (OH•), lipid radical (L•), lipid peroxyl radical (LOO•), alkoxyl (LO•), and peroxyl radicals [27]. 

GPE showed significant antioxidant activity against various radicals. We were able to build a dose-response curve for GPE using all of the methodologies employed, demonstrating that these methods were adequate to evaluate the antioxidant activity of GPE. The IC_50_ values were 0.009 *μ*L/mL, 0.070 *μ*L/mL, 0.003 *μ*L/mL, and 0.002 *μ*L/mL for the lipid peroxidation assay, the H_2_O_2_/luminol/HRP assay, the xanthine/luminol/XOD assay, and the deoxyribose assay, respectively (Table 1). The same *in vitro* methodologies were previously used by our group [15] to determine the IC_50_ values of BPE. 

In an attempt to observe which free radicals were efficiently scavenged by each propolis extract, the IC_50_ values were compared. BPE achieved the lowest IC_50_ value for the xanthine/luminol/XOD system, suggesting better activity against superoxide radicals than against the other radicals. In comparison, GPE showed the lowest IC_50_ value for the inhibition of the degradation of the deoxyribose sugar, suggesting better activity against hydroxyl radicals than against the other radicals.

### 3.3. In Vivo Protective Effect against UVB-Induced Oxidative Stress

Based on the evidence for antioxidant activities described above, the propolis extracts were evaluated *in vivo* against UVB-induced oxidative stress. There was a dose-dependent depletion of GSH in the skin of hairless mice after UV irradiation [20]. In the irradiated control animals (Group 2), the dose of irradiation used (2.46 J/cm^2^) induced a decrease of 47.3% and 41.0% in the GSH level for the topical and oral treatments, respectively, when compared to the nonirradiated control animals (Group 1).

**Figure 2:**
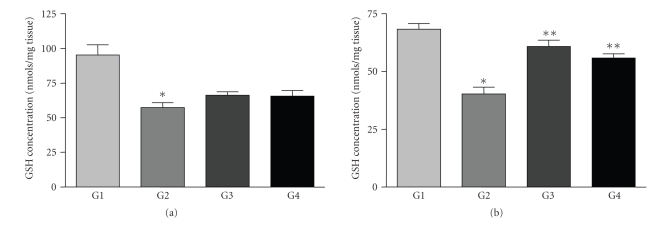
*In vivo *protective effect as assessed by the GSH depletion assay. G1 = nonirradiated control, G2 = irradiated control, G3 = GPE, and G4 = BPE. (a) Topical treatment and (b) Oral treatment. Bars represent means ± SE of three replicates. Statistical analysis was performed using the Student's *t*-test. *P < 0.05 compared to the nonirradiated control and **P < 0.05 compared to the irradiated control.

Topical pretreatment of animals with solutions containing both propolis extracts (Groups 3 and 4) led to a 14.0% recovery GSH levels after irradiation (Figure 2(a)). However, there was no significant difference (*P* = 0.112 for GPE and *P* = 0.194 for BPE) between these groups of animals and the nontreated ones (Group 2). 

In contrast to the results obtained with topical treatment, the oral administration of BPE and GPE demonstrated potential effects against oxidative stress. Pretreatment with GPE and BPE solutions (Groups 3 and 4) significantly (*P* = 0.001) prevented UV-induced GSH depletion when compared to nontreated irradiated animals (Group 2), leading to a recovery of 30.0% by GPE and 22.8% by BPE in GSH levels (Figure 2(b)). There was no significant difference between the potential *in vivo *protective effects of the two extracts. 

The ability of the propolis extracts to inhibit proteinase secretion/activity induced by UVB irradiation was also investigated in this study. Enzymography of the gelatinases (MMP-2 and MMP-9) in the skin showed that MMP-9 only appeared in irradiated skin, as observed in previous studies performed by our group [19, 20]. It was visually observed that neither topical nor oral treatments with propolis extracts inhibited the increase in cutaneous metalloproteinase-9 activity induced by exposure to UV irradiation (data not shown). In addition, gelatinase-2 (MMP-2) was present in all groups of irradiated animals and nonirradiated animals. Neither topical nor oral treatments containing propolis extracts altered cutaneous metalloproteinase-2 activity (data not shown).

## 4. Discussion

The present study evaluated, two different marketed propolis extracts: brown and green. Brown propolis is the most common propolis worldwide, and the extract used in this study was a blend of propolis collected in several regions in Brazil. In contrast, green propolis is found only in the southern region of Brazil, where the main plant source of propolis is *Baccharis sp.* The green propolis found in this area is rich in prenylated derivatives of *p*-coumaric acid, such as Artepillin C [28, 29].

It can be inferred from the results that the GPE contained greater amounts of polyphenols but had lesser amounts of flavonoids compared to BPE. In addition, the polyphenol content in BPE was about 2.8 times that of the flavonoid content, while the polyphenol content in GPE was 7.8 times that of the flavonoid content. The flavonoid and polyphenol contents measured in this study were very close to those previously reported in marketed Brazilian propolis extracts from several regions; flavonoid content ranged from 0.05% to 0.7% and polyphenol content from 0.4% to 3.9% in previous reports [30].

Although both extracts demonstrated considerable antioxidant activity, GPE's activity was significantly more efficient than BPE's in all of the tested systems. This difference between the extracts' antioxidant activities may be due to qualitative and quantitative differences in their physico-chemical compositions. It is well established that phenolic compounds, such as the ones present in propolis, work as antioxidants by breaking the chain reaction of lipids [31], inhibiting chemiluminescence reactions [32], scavenging several ROS [33], and so forth. Nevertheless, no correlation between phenolic and flavonoid contents and antioxidant activity has been confirmed. Until now, reports have suggested that antioxidant properties arise from complex mechanisms or synergistic interactions between compounds [34, 35].

The results obtained in this study revealed that antioxidant activity was mainly linked to the polyphenol content of the sample and was less dependent upon the flavonoid content. This suggestion is based on the observation that the concentration of flavonoids in BPE was significantly higher than that in GPE, but BPE showed the lower antioxidant activity. In addition, it can be suggested that marker compounds (*p*-coumaric acid, drupanin, artepillin C, and baccharin) may contribute to the elevated antioxidant activity of GPE.

Quercetin, a flavonoid with well-known antioxidant activity, is used as a reference antioxidant compound to evaluate the activity of different extracts. As demonstrated by Vicentini et al. (2007) [36], quercetin showed an IC_50_ of 0.2 *μ*g/mL for the inhibition of lipid peroxidation and an IC_50_ of 1.05 *μ*g/mL for the H_2_O_2_/luminol/HRP assay. For the xanthine/luminol/XOD assay, quercetin achieved an IC_50_ value of 11.3 *μ*g/mL [19]. By comparing, the IC_50_ values found for propolis extracts with those obtained for quercetin, it can be concluded that BPE was effective in scavenging superoxide radicals produced in the xanthine/luminol/XOD system. GPE performed well when scavenging hydroxyl radicals, and quercetin was effective against the hydroxyl, peroxyl, and alkoxyl radicals produced during lipid peroxidation. Besides this, GPE showed higher antioxidant activity when compared with *ginkgo biloba* and isoflavin beta [35].

The *in vitro* antioxidant activity studies demonstrated the suitable applicability of these extracts against free radicals, leading the group to research their potential *in vivo* protective effects. The oral treatment of hairless mice with both extracts prevented irradiation-induced oxidative stress by preventing GSH depletion. However, topical pretreatment of animals with solutions containing both propolis extracts was not effective against UV irradiation-induced GSH depletion.

The low topical effectiveness of both extracts could be explained by poor diffusion of the antioxidant compounds through the stratum corneum and viable epidermis of mouse skin. Therefore, increasing the diffusion of antioxidant compounds through the skin might be an avenue to better assess the real potential of propolis against oxidative stress in skin. To this end, topical formulations and/or enhancer promoters could be employed, as reported by Marquele et al. [37] for topical formulations with propolis extract and Casagrande et al. [20] and Vicentini et al. [23] for topical treatments with quercetin.

The protective effect achieved by oral administration of both extracts could be explained by the absorption of compounds present in these extracts by the intestinal tract and their subsequent distribution in the blood to several organs, including the skin. Furthermore, oral administration of antioxidants could protect the entire skin surface without being affected by washing, perspiration, or rubbing, all of which could lessen the efficacy of topical applications. Additionally, the effectiveness of topical applications could be limited by poor diffusion of antioxidant compounds into the epidermis [38]. 

It is clear that GPE and BPE presented similar protection against oxidative stress, as assessed by GSH protection. This observation corroborates the finding of Bankova [39], who reported that propolis extracts with different physico-chemical compositions, but with the same dry weight, showed similar biological properties. The authors suggested that even though different propolis extracts had similar antioxidant activities, the compounds responsible for these activities in each extract could be different.

The protective effect achieved by oral treatment with propolis extracts against UVB irradiation-induced GSH depletion was similar to that observed by our group through the oral treatment of hairless mice with a marigold extract [19] and by topical treatment of hairless mice with a quercetin-loaded microemulsion [23].

**Figure 3:**
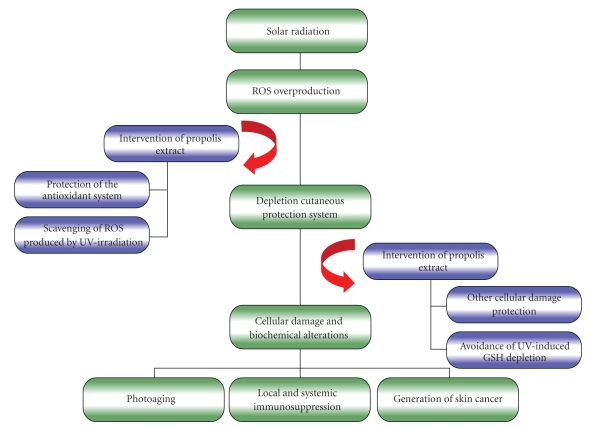
Hypothetical diagram of UV irradiation-induced skin damages and intervention of propolis extract. Degenerative processes related to UV irradiation skin exposure are largely mediated by the overproduction of reactive oxygen species (ROS) and by the impairment of antioxidant systems. Thus, the ROS not eliminated by the biological system can cause cellular damage and biochemical alterations, such as oxidation of proteins and lipids, inflammation, damage to DNA, and activation and inactivation of enzymes. These biochemical alterations generate clinical signals as photoaging, local and systemic immunosuppression, and skin cancer. We showed that propolis extract may interfere in the overproduction of ROS and in the UV irradiation-induced GSH depletion. Then, the present study suggests the potential applicability of propolis extracts against UV-induced skin damages.

The GPE effect is probably due to inhibition of oxidative stress as described by Cole et al. [40], who has demonstrated that the topical application of “Sydney” propolis reduced cutaneous inflammation, immunosuppression, and lipid peroxidation induced by UV exposure (Figure 3). Moreover, it is important to consider the possibility of UV absorption by propolis (photoprotective effect) as it has been reported by Soares dos Reis et al. [41], who demonstrated by *in vitro* analyses considerable values of sun protector factor (FPS) for formulations with hidroalcoholic extract of green propolis.

While both extracts prevented UV irradiation-induced GSH depletion, neither extract demonstrated the capacity to inhibit metalloproteinase activity. However, *in vitro* studies have shown that propolis inhibits metalloproteinase activity in tumor cell cultures. Jin et al. [42] demonstrated that propolis extracts in ethanol strongly inhibited MMP-9 activity in a concentration-dependent manner in the hepatocarcinoma cell line Hep3B. 

When taking these studies into consideration, the lack of positive results for both extracts in this study could be due to the physiological mechanisms involved in the upregulation of MMPs after UV irradiation. It is possible that the regulation of MMPs in normal cells after UV irradiation might be different from the regulation of MMPs in cancerous cells. Additionally, the effect of propolis compounds in *in vitro* cancerous cell culture might be different from the effects of propolis compounds in normal cells in the *in vivo* system used in this study. As was previously mentioned, the capacity of the antioxidant compounds to reach the “target tissue” of mice skin could limit the extract' ability to regulate MMPs.

## 5. Conclusion

The present study suggests the potential applicability of propolis extracts for preventing UV-induced skin damages. Both BPE and GPE extracts exhibited considerable antioxidant activity and inhibited UV irradiation-induced GSH depletion, and the oral treatments were more effective than the topical treatments. Despite differences in their physico-chemical composition and *in vitro* antioxidant activities, both extracts showed similar *in vivo *effects against oxidative stress in skin by protecting against GSH depletion. To cultivate a more complete understanding of the protective capabilities of these extracts, topical formulations that diffuse more effectively through the skin could be developed and additional studies be performed.

## Figures and Tables

**Table 1:** Physico-chemical composition and antioxidant activity of BPE and GPE.

Physico-chemical composition (*μ*g/mL)	BPE	GPE
*p*-coumaric acid	74.6	275.7
Drupanin	213.6	529.5
Artepillin C	477.9	1053.7
Baccharin	585.1	1060.8

Antioxidant activity (IC_50_ –*μ*L/mL)	BPE	GPE

Lipid peroxidation assay	0.016*	0.009
H_2_O_2_/luminol/HRP assay	0.221*	0.070
Xanthine/luminol/XOD assay	0.005*	0.003
Deoxyribose assay	0.024*	0.002

*IC_50_ values determined by Marquele et al. [15]

Results are represented by mean of 3 determinations.

## References

[1] Shindo Y, Witt E, Packer L (1993). Antioxidant defense mechanisms in murine epidermis and dermis and their responses to ultraviolet light. *Journal of Investigative Dermatology*.

[2] Bonina F, Lanza M, Montenegro L (1996). Flavonoids as potential protective agents against photo-oxidative skin damage. *International Journal of Pharmaceutics*.

[3] Atoui AK, Mansouri A, Boskou G, Kefalas P (2005). Tea and herbal infusions: their antioxidant activity and phenolic profile. *Food Chemistry*.

[4] Montenegro L, Bonina F, Rigano L, Giogilli S, Sirigu S (1995). Protective effect evaluation of free radical scavengers on UVB induced human cutaneous erythema by skin reflectance spectrophotometry. *International Journal of Cosmetic Science*.

[5] Díaz-Carballo D, Malak S, Bardenheuer W, Freistuehler M, Reusch HP (2008). The contribution of plukenetione A to the anti-tumoral activity of Cuban propolis. *Bioorganic and Medicinal Chemistry*.

[6] Paulino N, Abreu SRL, Uto Y (2008). Anti-inflammatory effects of a bioavailable compound, Artepillin C, in Brazilian propolis. *European Journal of Pharmacology*.

[7] Moreira L, Dias LG, Pereira JA, Estevinho L (2008). Antioxidant properties, total phenols and pollen analysis of propolis samples from Portugal. *Food and Chemical Toxicology*.

[8] Benkovic V, Knezevic AH, Dikic D (2008). Radioprotective effects of propolis and quercetin in *γ*-irradiated mice evaluated by the alkaline comet assay. *Phytomedicine*.

[9] Bhadauria M, Nirala SK, Shukla S (2008). Multiple treatment of propolis extract ameliorates carbon tetrachloride induced liver injury in rats. *Food and Chemical Toxicology*.

[10] Sforcin JM (2007). Propolis and the immune system: a review. *Journal of Ethnopharmacology*.

[11] Scazzocchio F, D’Auria FD, Alessandrini D, Pantanella F (2006). Multifactorial aspects of antimicrobial activity of propolis. *Microbiological Research*.

[12] Kumazawa S, Hamasaka T, Nakayama T (2004). Antioxidant activity of propolis of various geographic origins. *Food Chemistry*.

[13] Simões LMC, Gregório LE, Da Silva Filho AA (2004). Effect of Brazilian green propolis on the production of reactive oxygen species by stimulated neutrophils. *Journal of Ethnopharmacology*.

[14] Souza JPB, Furtado NAJC, Jorge R, Soares AEE, Bastos JK (2007). Perfis fisico-quimico e cromatogrfico de amostras de propolis produzidas nas microrregioes de Franca (SP) e Passos (MG), Brasil. *Revista Brasileira de Farmacognosia*.

[15] Marquele FD, Di Mambro VM, Georgetti SR, Casagrande R, Lucisano-Valim YM, Fonseca MJV (2005). Assessment of the antioxidant activities of Brazilian extracts of propolis alone and in topical pharmaceutical formulations. *Journal of Pharmaceutical and Biomedical Analysis*.

[16] Krol W, Czuba Z, Scheller S, Paradowski Z, Shani J (1994). Structure-activity relationship in the ability of flavonols to inhibit chemiluminescence. *Journal of Ethnopharmacology*.

[17] Girotti S, Fini F, Ferri E, Budini R, Piazzi S, Cantagalli D (2000). Determination of superoxide dismutase in erythrocytes by a chemiluminescent assay. *Talanta*.

[18] Halliwell B, Gutteridge JMC, Aruoma OI (1987). The deoxyribose method: a simple “test-tube” assay for determination of rate constants for reactions of hydroxyl radicals. *Analytical Biochemistry*.

[19] Fonseca YM, Catini CD, Vicentini FTMC, Nomizo A, Gerlach RF, Fonseca MJV (2010). Protective effect of Calendula officinalis extract against UVB-induced oxidative stress in skin: evaluation of reduced glutathione levels and matrix metalloproteinase secretion. *Journal of Ethnopharmacology*.

[20] Casagrande R, Georgetti SR, Verri WA, Dorta DJ, dos Santos AC, Fonseca MJV (2006). Protective effect of topical formulations containing quercetin against UVB-induced oxidative stress in hairless mice. *Journal of Photochemistry and Photobiology B*.

[21] Hissin PJ, Hilf R (1976). A fluorometric method for determination of oxidized and reduced glutathione in tissues. *Analytical Biochemistry*.

[22] Onoue S, Kobayashi T, Takemoto Y, Sasaki I, Shinkai H (2003). Induction of matrix metalloproteinase-9 secretion from human keratinocytes in culture by ultraviolet B irradiation. *Journal of Dermatological Science*.

[23] Vicentini FTMC, Simi TRM, Del Ciampo JO (2008). Quercetin in w/o microemulsion: *in vitro* and *in vivo* skin penetration and efficacy against UVB-induced skin damages evaluated *in vivo*. *European Journal of Pharmaceutics and Biopharmaceutics*.

[24] Lowry OH, Rosebrough NJ, Farr AL, Randall RJ (1951). Protein measurement with the Folin phenol reagent. *The Journal of Biological Chemistry*.

[25] Podda M, Traber MG, Weber C, Yan L-J, Packer L (1998). UV-irradiation depletes antioxidants and causes oxidative damage in a model of human skin. *Free Radical Biology and Medicine*.

[26] Marquele-Oliveira F, Fonseca YM, de Freitas O, Fonseca MJV (2007). Development of topical functionalized formulations added with propolis extract: stability, cutaneous absorption and *in vivo* studies. *International Journal of Pharmaceutics*.

[27] Tedesco AC, Martínez L, González S (1997). Photochemistry and photobiology of actinic erythema: defensive and reparative cutaneous mechanisms. *Brazilian Journal of Medical and Biological Research*.

[28] Park YK, Paredes-Guzman JF, Aguiar CL, Alencar SM, Fujiwara FY (2004). Chemical constituents in *Baccharis dracunculifolia* as the main botanical origin of southeastern brazilian propolis. *Journal of Agricultural and Food Chemistry*.

[29] Salatino A, Teixeira ÉW, Negri G, Message D (2005). Origin and chemical variation of Brazilian propolis. *Evidence-Based Complementary and Alternative Medicine*.

[30] da Silva JFM, de Souza MC, Matta SR, de Andrade MR, Vidal FVN (2006). Correlation analysis between phenolic levels of Brazilian propolis extracts and their antimicrobial and antioxidant activities. *Food Chemistry*.

[31] Torel J, Cillard J, Cillard P (1986). Antioxidant activity of flavonoids and reactivity with peroxy radical. *Phytochemistry*.

[32] Georgetti SR, Casagrande R, Di Mambro VM, Azzolini AE, Fonseca MJ (2003). Evaluation of the antioxidant activity of different flavonoids by the chemiluminescence method. *AAPS PharmSci*.

[33] Bors W, Heller W, Michel C, Saran M (1990). Flavonoids as antioxidants: determination of radical-scavenging efficiencies. *Methods in Enzymology*.

[34] Ramanauskiene K, Kalveniene Z, Kasparaviciene G, Briedisis V (2007). Assay of correlation between antioxidant activity of propolis extracts and their chemical components. *European Journal of Pharmaceutical Sciences*.

[35] Marquele-Oliveira F, Fonseca YM, Georgetti SR, Vicentini FTMC, Bronzati V, Fonseca MJV (2008). Evaluation of the antioxidant activity as an additional parameter to attain the functional quality of natural extracts. *Latin American Journal of Pharmacy*.

[36] Vicentini FTMC, Casagrande R, Georgetti SR, Bentley MVLB, Fonseca MJV (2007). Influence of vehicle on antioxidant activity of quercetin: a liquid crystalline formulation. *Latin American Journal of Pharmacy*.

[37] Marquele FD, Oliveira ARM, Bonato PS, Lara MG, Fonseca MJV (2006). Propolis extract release evaluation from topical formulations by chemiluminescence and HPLC. *Journal of Pharmaceutical and Biomedical Analysis*.

[38] González S, Fernández-Lorente M, Gilaberte-Calzada Y (2008). The latest on skin photoprotection. *Clinics in Dermatology*.

[39] Bankova V (2005). Recent trends and important developments in propolis research. *Evidence-Based Complementary and Alternative Medicine*.

[40] Cole N, Sou PW, Ngo A (2010). Topical ‘sydney’ propolis protects against UV-radiation-induced inflammation, lipid peroxidation and immune suppression in mouse skin. *International Archives of Allergy and Immunology*.

[41] Soares dos Reis G, Furtado Valadao A, Ramos Paes de Lima L, Lucy Moreira M (2009). Preparacion de um protector solar y evaluacion de La accion fotoprotetora Del propoleo verde Del Vale do Aco, Minas Gerais, Brasil. *Boletin Latinoamericano y Del Caribe de Plantas Medicinales y Aromaticas*.

[42] Jin U-H, Chung T-W, Kang S-K (2005). Caffeic acid phenyl ester in propolis is a strong inhibitor of matrix metalloproteinase-9 and invasion inhibitor: isolation and identification. *Clinica Chimica Acta*.

